# Update on Diagnosis and Treatment of Diabetic Retinopathy: A Consensus Guideline of the Working Group of Ocular Health (Spanish Society of Diabetes and Spanish Vitreous and Retina Society)

**DOI:** 10.1155/2017/8234186

**Published:** 2017-06-14

**Authors:** Borja Corcóstegui, Santiago Durán, María Olga González-Albarrán, Cristina Hernández, José María Ruiz-Moreno, Javier Salvador, Patricia Udaondo, Rafael Simó

**Affiliations:** ^1^Spanish Retina and Vitreous Society (SERV), IMO (Institut Microcirurgia Ocular), Barcelona, Spain; ^2^Spanish Society of Diabetes (SED), Endocrinology Department, Valme University Hospital and RAMSE Foundation, Sevilla, Spain; ^3^Spanish Society of Diabetes (SED), Endocrinology and Nutrition Department, Hospital General Universitario Gregorio Marañón, Madrid, Spain; ^4^Spanish Society of Diabetes (SED), Diabetes and Metabolism Research Unit and CIBERDEM (ISCIII), Vall d'Hebron Reseach Institute (VHIR), Barcelona, Spain; ^5^Spanish Retina and Vitreous Society (SERV), Department of Ophthalmology, Castilla La Mancha University, Albacete, Spain; ^6^University Hospital Puerta de Hierro-Majadahonda, Madrid, Spain; ^7^Spanish Society of Diabetes (SED), Department of Endocrinology and Nutrition, University Clinic of Navarra, Pamplona, Spain; ^8^Spanish Retina and Vitreous Society (SERV), Department of Ophthalmology, Nuevo Hospital Univeristario y Politécnico La Fe, Valencia, Spain

## Abstract

A group of members of the Spanish Retina and Vitreous Society (SERV) and of the Working Group of Ocular Health of the Spanish Society of Diabetes (SED) updated knowledge regarding the diagnosis and treatment of diabetic retinopathy (DR) based on recent evidence reported in the literature. A synthesis of this consensus forms the basis of the present review, which is intended to inform clinicians on current advances in the field of DR and their clinical applicability to patients with this disease. Aspects presented in this article include screening procedures of DR, new technologies in the early diagnosis of DR, control of risk factors in the different stages of the disease, indications of panretinal laser photocoagulation, efficacy of intravitreal antiangiogenic agents and steroids, and surgical options for treating DR-related complications. Practical information regarding periodicity of screening procedures in patients with type 1 and type 2 diabetes, ophthalmological controls according to the stage of retinopathy and complications, and criteria and degree of urgency for referral of a DR patient to the ophthalmologist are also presented.

## 1. Introduction

According to the International Federation of Diabetes (IFD), there will be 642 million people with diabetes in the world in 2040, with a foreseeable dramatic burden of the disease, particularly worrisome in the most extreme population segments, that is, the young people and the elderly subjects [1]. These alarming data have an even greater impact on the possible effects of the numerous complications resulting from diabetes. From a traditional perspective, chronic complications of diabetes have been classified into microangiopathic or diabetes-specific (retinopathy, nephropathy, and neuropathy) and macroangiopathic often regarded as equivalent to atheromatosis. The three microvascular complications of diabetes show a complex interrelationship [2]. Also, microvascular and macrovascular complications frequently coexist [3].

The causative role of hyperglycemia in the development of complications is well established. Classical studies, such as the Diabetes Control and Complications Trial (DCCT) [4] and the United Kingdom Diabetes Study (UKPDS) [5] showed that early strict glycemic control, both in type 1 and type 2 diabetes, can delay the onset and progression of microvascular complications. However, in addition to hyperglycemia, other factors, such as hypertension, dyslipidemia, hemorrheologic changes, and particularly the genetic load, have a remarkable influence on the severity and clinical course of diabetic retinopathy (DR).

In this paper, a panel of members of the Working Group of Ocular Health, which consists of expert members belonging to the Spanish Retina and Vitreous Society (BC, JMRM, and PU) and the Spanish Society of Diabetes (SD, MOGA, CR, JS, and RS) summarized the main conclusions of a workshop aimed at creating a consensus regarding the pathophysiology, diagnosis, and treatment of diabetic retinopathy (DR) based on recent evidence reported in the literature.

### 1.1. Pathophysiology of DR and Diabetic Macular Edema

DR is the most frequent microvascular complication, the prevalence of which increases with the duration of diabetes, with an overall rate of up to 30% and a high risk of severe visual impairment in 10% of subjects [6]. Diabetic macular edema (DME) is more frequent in type 2 diabetes, occurs in approximately 7.5% of diabetic patients, and is the main cause of blindness in working-age adults in industrialized countries [7].

**Figure 1:**
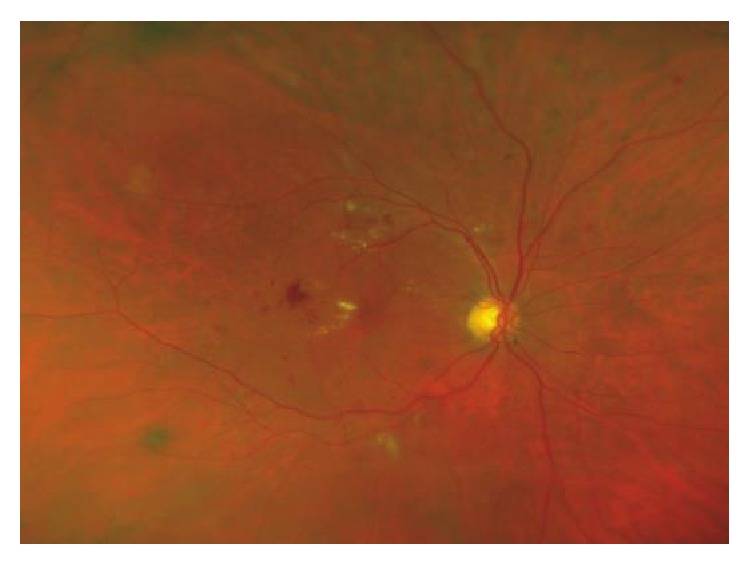
Nonproliferative diabetic retinopathy showing microaneurysms, microhemorrhages, and hard exudates.

Elevated blood glucose levels per se and the metabolic pathways directly related to hyperglycemia, such as the polyol and hexosamine pathways, activation of the diacylglycerol-protein kinase C pathway, and accumulation of advanced glycation end products, are involved in the pathophysiology of DR [8]. Inflammation, alteration of retinal blood flow autoregulation, and hemorrheological factors also play an important role in the pathogenesis of DR [8]. Thickening of the basement membrane, pericyte loss, and disruption of interendothelial tight junctions are characteristic pathophysiological mechanisms in early stages of DR. Microaneurysm formation and fluid extravasation from the intravascular to the interstitial space can lead to retinal thickening and hard exudates [8]. This first stage is called nonproliferative diabetic retinopathy (NPDR), or the so-called background DR (Figure 1).

**Figure 2:**
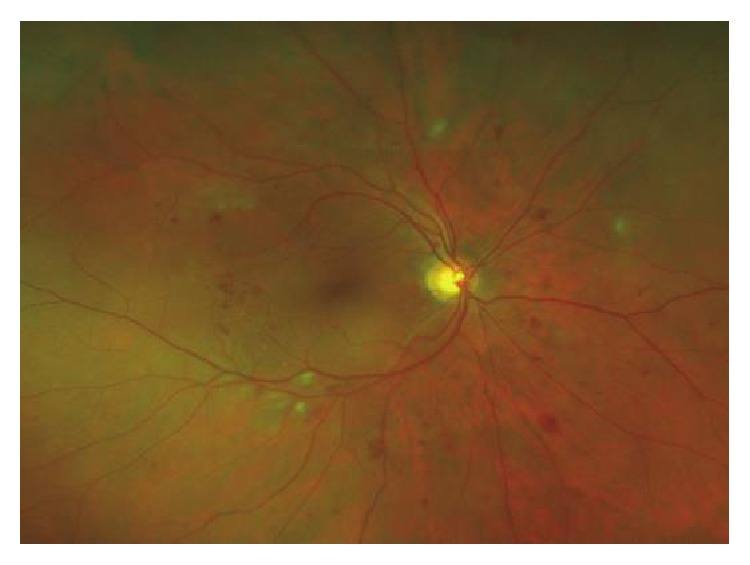
Proliferative diabetic retinopathy showing the presence of neovascularization.

Loss of the capillary endothelium, thrombus formation, retinal leukostasis, and complete occlusion of the capillary lumen appear at later stages of the disease. Cotton-wool spots or soft exudates, reflecting infarct zones and intraretinal microcirculatory alterations, are hallmark features of preproliferative DR [9] (Figure 2).

**Figure 3:**
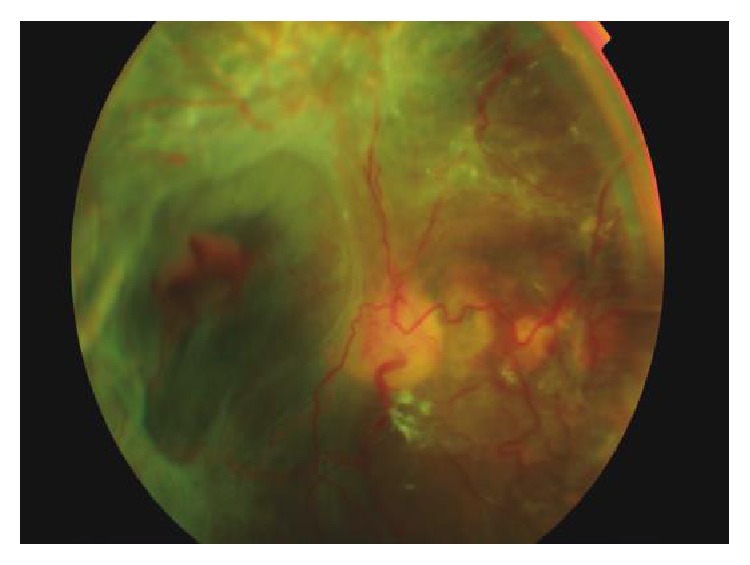
Proliferative diabetic retinopathy and tractional retinal detachment caused by fibrovascular tissue.

Basement membrane digestion by proteolytic enzymes is essential for angiogenesis (neovascularization). Degradation products and hypoxia are potent activators of angiogenesis. Hypoxia promotes vessel growth by upregulating multiple proangiogenic pathways, particularly the vascular endothelial growth factor (VEGF), which plays a pivotal role in the development of pathologic angiogenesis [10]. This stage known as proliferative retinopathy (PDR) is characterized by growth of new vessels (Figure 3). The new vessels attached to the posterior hyaloid become fibrotic and may cause tractional retinal detachment. Vitreous hemorrhage may result from fragility and bleeding of neovascular vessels [9].

Rupture of the inner or the outer blood retinal barriers leading to extravasation of the intravascular content and increased intravascular colloid osmotic pressure are early events in the pathogenesis of diabetic macular edema (DME). Proinflammatory cytokines and VEGF are involved in the breakdown of blood-retinal barrier [11].

There is growing evidence suggesting that retinal neurodegeneration is an early event in the pathogenesis of DR, which participates in the development of microvascular abnormalities [12]. This progressive degenerative process is characterized by neural apoptosis and reactive gliosis. Retinal neurodegeneration causes functional alterations, such as loss of color discrimination and reduced contrast sensitivity. Electrophysiological evaluation is the most sensitive method for detecting neurodegeneration. It is worth mentioning that electrophysiological abnormalities can appear even before that any impairment can be detected in the fundoscopic examination. Also, treatment based on neuroprotection opens up a new approach for preventing or arresting DR development [13].

### 1.2. Classification

**Figure 4:**
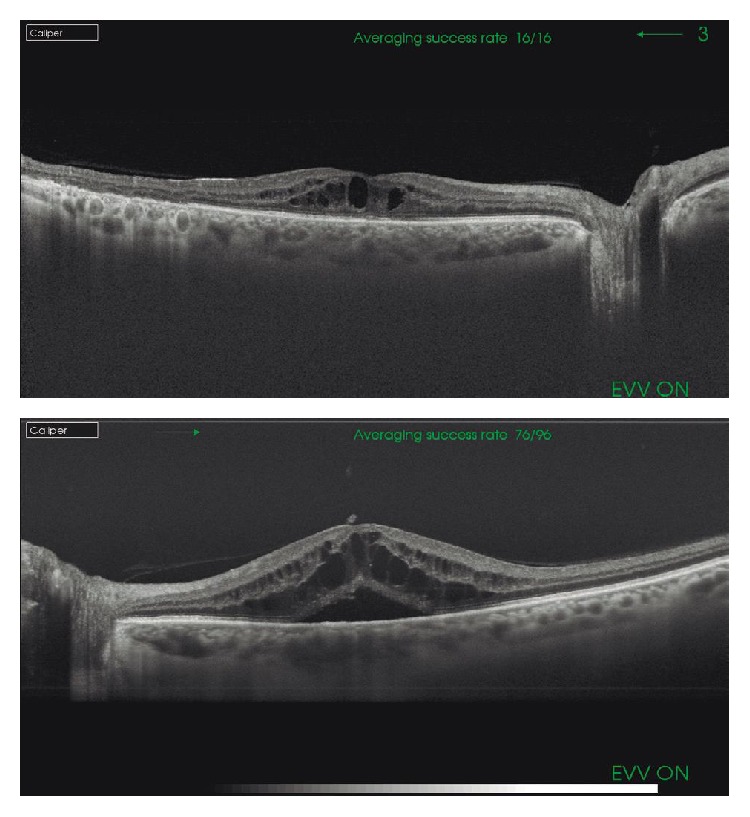
Diabetic macular edema (DME). (a) We can observe the presence of DME with intraretinal cysts. (b) Apart from cysts, neuroretinal detachment can be noticed (SS-OCT images).

Definitions used in the Early Treatment Diabetic Retinopathy Study (ETDRS) [14, 15] have provided uniform criteria for the terminology and classification of DR and DME, which have been included in the 2016 preferred practice pattern guidelines for DR issued by the American Academy of Ophthalmology [16]. DR is classified into two basic stages: NPDR and PDR. NPDR is divided into mild, moderate, and severe according to disease severity level. DME is defined as apparently absent and apparently present. It is important to remember that visual acuity (VA) is not included in the definition of DME. Clinically significant DME is present when the following three criteria are met: retinal thickening at or within 500 *μ*m of the center of the macula; hard exudates at or within 500 *μ*m of the center of the macula, if associated with thickening of the adjacent retina; and/or a zone (or zones) of retinal thickening one disc area in size at least part of which is within one disc diameter of the center [16]. According to the morphology of the macula on OCT, DME is divided into three groups: spongiform, cystoid, and neuroepithelial retinal detachment (Figure 4). Fundus fluorescein angiography identifies focal (or multifocal), diffuse, ischemic, and mixed DME.

In recent years, OCT has revolutionized the diagnoses and monitoring of DME, thus facilitating its management (see Section 2.3).

## 2. Prevention of DR

### 2.1. Risk Factors

Duration of diabetes, poor control of blood glucose, and hypertension are major risk factors for rapid progression of RD [8]. A rapid lowering of blood glucose levels [17, 18] and hypoglycemia [19] may aggravate preproliferative DR and precipitate vitreous hemorrhage in patients with PDR. Insulin-dependent type 1 diabetes is associated with a higher risk of DR and severe forms (PDR) as compared with type 2 diabetes. The percentages of DR may vary from 85% for insulin-dependent patients to 58% for non-insulin-dependent patients for more than 15 years after diagnosis [20]. Dyslipidemia [21], puberty [22], pregnancy [23], diabetic nephropathy [24, 25], and obesity [26] have also been reported as risk factors for DR.

Tight metabolic control, control of risk factors, and close monitorization of progression of preexisting DR are indispensable measures to maximally prevent vision loss. A recommended approach for the control of patients with RD is shown in Table 1.

### 2.2. Screening for DR

Early diagnosis of DR is the best strategy to prevent or delay loss of vision. Although regular fundus examination is widely recommended in screening protocols for early treatment of retinal lesions prior to the appearance of visual difficulties, different studies have shown that, in daily practice, only a small percentage of diabetic subjects underwent fundus exam with the recommended periodicity [27]. Direct ophthalmoscopy requires pupillary dilation and skills for the procedure. However, general practitioners can screen for DR with a high level of accuracy using nonmydriatic retinography. This cost-effective diagnostic tool is used to obtain digital photographs of the retina (retinographies), which can be stored in the computer and efficiently send by the family physician to the ophthalmologist for assessment. Although general practitioners should play a pivotal role in the screening of DR, this will depend on their skillfulness in performing fundus examination (Table 1), as well as the availability of nonmydriatic retinal cameras.

Different studies have shown that the frequency of screening tests can be modified according to the stage of DR. In a dynamic cohort study of 20,686 people with type 2 diabetes who had annual retinal photography up to 14 times between 1990 and 2006, after 5 years of follow-up, few patients without retinopathy at baseline developed preproliferative retinopathy or sight-threatening maculopathy, whereas patients with NPDR at baseline were five times more likely to develop preproliferative, PDR, or maculopathy [28]. Screening intervals at 2 years may be appropriate for subjects without DR at the initial screening examination. Other studies have also shown that the strategy of biannual screening is safe and cost-effective for subjects who have not developed DR [29, 30].

The periodicity of screening for DR is summarized in Table 2. In type 1 diabetes, beginning of screening is recommended after 5 years of diagnosis and in people older than 15 years. Subjects with type 2 diabetes should start screening immediately after diagnosis and before the end of the first trimester in pregnant women with diabetes. The periodicity of screening is recommended annually except for type 2 diabetes without signs of DR with adequate metabolic control and short duration of the disease.

Recommendations of ophthalmological examinations in patients with DR according to complications and stages of DR are shown in Table 3. In patients with the presence of central-involved DME (CIDME), or edema affecting the 1 mm in diameter retinal central subfield, intravitreous therapy with a careful follow-up every 1–4 months is recommended. When non-CIDME is present, controls should be scheduled every 6 months in mild NPDR and every 3-4 in moderate and severe NPDR. In the absence of DME, patients should be visited annually when NPDR is mild, every 6–12 months if NPDR is moderate, and every 6 months if NPDR is severe. Patients with PDR should be controlled at 3-month intervals. Apart from ophthalmological examinations, a tight control of blood glucose levels, blood pressure, and serum lipids are recommended.

The criteria adopted by the panel for either general screening follow-up of patients in whom DR was already diagnosed are similar to other guidelines such as the recently reported position statement of the American Diabetes Association [31].

Finally, criteria and level of urgency according to ophthalmologic findings for referral of patients with DR to the ophthalmologist are detailed in Table 4.

### 2.3. Early Diagnosis of DR

**Figure 5:**
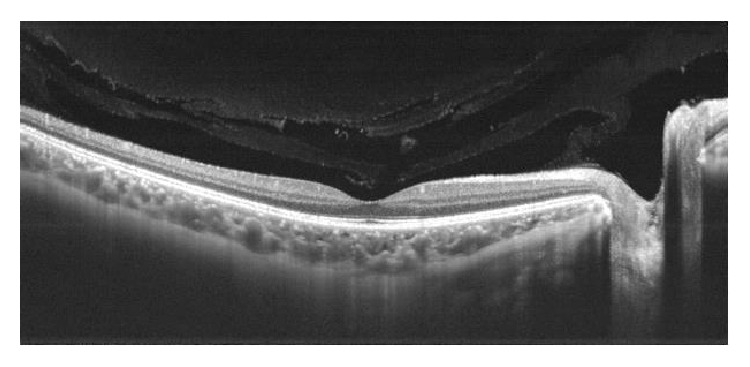
OCT in healthy patient showing in high resolution the different structures: choroid, retina layers, and vitreous gel.

Ophthalmoscopy with or without the pupil dilated is the standard procedure in the screening for DR, in which detection of microaneurysms in the posterior pole is the earliest clinical sign [32, 33]. Fluorescein angiography is an invasive, costly, and time-consuming technique but is a sensitive method to detect vascular changes due to rupture of the inner and outer blood retinal barrier in the course of an established DR [34]. In contrast to retinography or fluorescein angiograms, OCT provides high-resolution images of the retinal layers, choroid, vitreous gel, and the vitreoretinal interface and has become the gold standard for the diagnosis, treatment approach, prognosis, assessment of treatment response, and control of patients with DME (Figure 5). Because of the advantages of the speed and ease of image acquisition as compared to other examinations, the association of OCT to retinography may increase the sensitivity of early diagnosis/screening in the diabetic patient.

OCT angiography (OCTA) is a new noninvasive imaging technique that employs motion contrast imaging to high-resolution volumetric blood flow information generating images similar to angiographic images in a matter of seconds [35, 36]. It provides a highly detailed view of the retinal vasculature, which allows for accurate delineation of the foveal avascular zone (FAZ) and detection of subtle microvascular abnormalities, including FAZ enlargement, areas of capillary nonperfusion, and intraretinal cystic spaces [37]. The possibility of detecting microvascular changes in diabetic eyes before the presence of visible microaneurysms may have important implications in the future. As OCTA is fast and noninvasive, it can provide a sensitive method for detecting early changes in DR, constituting a very promising technique for early diagnosis and control of treatment in patients with DR [38–40]. In this sense, OCTA could be able to quickly identify diabetic individuals at risk for developing retinopathy, which in turn would require more frequent examinations and a higher optimization of metabolic control.

## 3. Treatment of DR

### 3.1. Control of Risk Factors in Different Stages of DR

Numerous studies have confirmed the relationship between glycemic control and DR as well as the efficacy of reduction of glycated hemoglobin (HbA1c) in the appearance and progression of DR. In patients with type 2 diabetes, the risk of diabetic complications is strongly associated with the degree of metabolic control. Each 1% reduction in HbA1c reduces any endpoint related to diabetes by 21% [41]. There is level 1 evidence (grade A recommendation) for intensive glycemic control for reducing the progression of DR [42, 43]. In the DCCT study of patients with type 1 diabetes, intensive therapy to maintain normal glucose blood levels and HbA1c < 6.5% reduced the risk for the development of retinopathy by 76% and the progression of retinopathy by 54% [4]. In patients with type 2 diabetes, results of the UKPDS study were similar [5]. In addition, this study showed that tight control of blood pressure was associated with a risk reduction of 34% in the proportion of patients with deterioration of retinopathy and 47% with deterioration in VA by three lines of ETDRS chart [44]. Also, in hypertensive patients with diabetes, a decrease in systolic blood pressure of 10 mmHg was associated with 35% reduction of the risk of progression of DR, 35% of the need of retinal photocoagulation, and a twofold reduction of the risk of vision loss. However, a very strict control of blood pressure (systolic blood pressure < 120 mmHg) did not show additional benefits [45].

The evidence regarding control of dyslipidemia and its effect on progression of DR is less than solid [46, 47]. However, the use of fenofibrate as a specific treatment for dyslipidemia has been associated with a reduction of the risk of progression of DR in clinical trials [48, 49]. Therefore, fenofibrate may have a relevant role in the prevention of DR in association with intensive treatment of traditional risk factors, such as hyperglycemia and hypertension [50, 51]. In addition to the lipid-modifying activity, fenofibrate has also numerous pleiotropic effects, which seem to have a more relevant role than the lipidic mechanisms in its beneficial effects on DR and DME [52].

In patients with DME, besides the control of risk factors identified for DR, a complete study of the renal function is recommended because of the well-established relationship between subclinical diabetic nephropathy (microalbuminuria/albuminuria) and the risk of DME [53].

A multidisciplinary approach including treatment of risk factors, particularly metabolic control and reduction of blood pressure, as well as the implementation of an adequate screening program seems the most effective intervention to prevent DR or to act on the early stages of retinopathy when AV is still unaffected.

### 3.2. Current Indications of Laser Photocoagulation

Once DR has been diagnosed, ophthalmological treatment with laser photocoagulation is especially directed to treat two key complications: retinal neovascularization and severe or clinically significant macular edema [31, 54].

Panretinal laser photocoagulation can be performed in a single or various sessions (availability of the laser equipment, severity of the retinopathy, patient's general condition, travel distance for treatment, etc.). In patients with regression of new vessels within the first 3 months of photocoagulation, the visual prognosis is usually excellent.

Treatment with panretinal photocoagulation is not indicated in mild and moderate NPDR [16] because the risk of progression to proliferative stages is very low. In patients with severe NPDR, the use of laser photocoagulation should be cautiously evaluated. It may be indicated in the presence of intraretinal signs suggestive of the development of PDR, such as venous beading, intraretinal microvascular abnormalities (IRMA), and an increasing number of microaneurysms and hemorrhages. On the other hand, early panretinal photocoagulation should be considered in those patients at a higher risk of progression, including patients with long-standing diabetes and poor metabolic control, presence of hypertension or advanced renal disease, noncompliance with scheduled visits, PDR in the fellow eye, cataracts with significant visual impairment limiting laser photocoagulation in the future, prior to cataract surgery, pregnancy or intention to become pregnant, and detection of generalized ischemic areas in the angiogram. In addition, laser treatment should be considered as an adjunctive therapy in eyes with persistent central-involved DME despite anti-VEGF therapy [31].

It is important to explain to the patient the following points: (a) panretinal photocoagulation can stop the progression of PDR, but not in all cases; (b) the risk of bleeding persists after treatment because the regression of neovascularization is slow; and (c) panretinal photocoagulation may produce a moderate decrease in vision, visual field or dark-adapted threshold, but the benefit far outweighs the side effects.

### 3.3. Current Treatment of DME: Role of Intravitreal Antiangiogenic Agents and Steroids

Intravitreal therapies with anti-VEGF agents, particularly aflibercept, ranibizumab, and bevacizumab, have substantially improved the prognosis of potentially severe ocular diseases, including DME. A recent report on the guidance for the management of DME has been recently published by the European Society of Retina Specialists [55].

Anti-VEGF treatment has superseded macular laser treatment and is now the first-line therapy for DME involving the central macula [56, 57]. Level 1 evidence from large, multicenter clinical trials has established the beneficial effect of anti-VEGF agents in patients with DME [49–55]. Intravitreal anti-VEFG treatment was associated with sustained EDTRS letter gains of best-corrected visual acuity (BCVA) and reduction of central retinal subfield thickness on OCT as compared to control groups (sham injections or laser photocoagulation) [58–64]. Treatment regimens after an initial load of intravitreal injections depend on each drug, but in the case of aflibercept, a regimen of 2 mg every 8 weeks (after five monthly doses) is a therapeutic option that can reduce substantially the number of intravitreal injections and visits and, consequently, the workload in ophthalmology practice [65]. In addition, there is a lower cost associated with fewer intravitreal injections. Also, up to one-third of eyes treated with aflibercept achieved a regression equal or greater than 2 steps in EDTRS score of the diabetic retinopathy severity scale (Diabetic Retinopathy Severity Score (DRSS)) at week 100, which should be considered not only a great achievement from a functional perspective but also a differential feature as compared to the remaining anti-VEGF drugs [62].

The DRCR.net Protocol T study [66] compared aflibercept, ranibizumab, and bevacizumab. The loading phase and subsequent flexible retreatment phase regimen were the same for all 3 study drugs. The interim results after 1 year showed a mean gain that was +2.1 letters higher for aflibercept 2 mg than for ranibizumab 0.3 mg (the approved dose in the US; 0.5 mg is the approved dose in Europe) (*p* = 0.03). Patients were monitored as often as every 4 weeks. A subgroup analysis showed that the superior effect of aflibercept was driven by the study participants with poorer baseline BCVA (<69 letters). Of a maximum possible number of injections of 13 in the first year, the aflibercept arm received a median of 9 injections; the bevacizumab and ranibizumab arms received a median of 10 injections. Intravitreal bevacizumab was inferior to both aflibercept and ranibizumab in most comparisons. Serious adverse event rates were comparable between study arms. The 2-year results [67] of the Protocol T study slightly changed this scenario. The difference in BCVA gain between aflibercept and ranibizumab for eyes with poorer baseline BCVA that was noted at 1 year decreased at 2 years. Nevertheless, the first-year behavior and the slightly better mean BCVA gain confirmed the superiority of aflibercept over ranibizumab in patients with poorer baseline BCVA when considering the area under the curve. It remains unclear if the 0.5 mg dose that is approved in Europe would have led to different results in the first year of Protocol T in favor of ranibizumab 0.5 mg.

It is worth mentioning that in the classical study of Aiello et al. [68] more than half of patients with PDR did not show increased VEGF levels in the vitreous fluid, which may explain why approximately 50% of patients with DME do not respond to anti-VEGF treatment. In this subgroup of patients, proinflammatory cytokines probably play a more relevant pathogenic role and intravitreal steroid injections may be a more plausible therapeutic option.

With regard to intravitreal steroids, there is level 1 evidence that intravitreal triamcinolone is inferior to laser treatment at 3-year follow-up [69]. Sustained corticosteroid delivery systems such as the dexamethasone delivery system (DDS) and the fluocinolone acetonide insert have both been approved for the treatment of DME. The DDS was originally approved for use in presudophakic eyes or phakic eyes scheduled to undergo cataract removal, but approval for the use in phakic eyes followed within months. Unfortunately, neither drug has been directly compared to anti-VEGF therapy in prospective, masked, randomized, multicenter trials. Visual acuity improvements for these sustained delivery systems average + 7 letters [70, 71], generally less than +8 to +12, achieved with anti-VEGF therapy [72]. The high rate of increased intraocular pressure (IOP) and cataract needs to be considered when using intravitreal steroid preparations. For these reasons, intraocular corticosteroids may be effective second-line therapy but are usually not used as first-line therapy. However, intravitral corticosteroids may be suitable for pseudophakic patients and in particular when chronic DME exists [69, 72, 73].

### 3.4. Intravitreal Antiangiogenic Agents for PDR Treatment

The Diabetic Retinopathy Clinical Research Network (DRCR.net) recently published the two-year results of Protocol S, which was designed as a noninferiority study to compare panretinal photocoagulation (PRP) and intravitreal ranibizumab (Lucentis, Genentech) for patients with high-risk PDR. Protocol S randomized eyes to receive one to three sessions of PRP treatment (203 eyes) or ranibizumab 0.5 mg intravitreal injection at baseline and then every four weeks (191 eyes). A structured retreatment protocol determined repeat injections based on SD-OCT and clinical findings. It is worth mentioning that eyes with DME received ranibizumab in both groups. The main findings were that vision outcomes and surgery rates were not inferior in the injection group. At 2 years, visual acuity improved by 2.8 letters from baseline in the ranibizumab group compared with an improvement of 0.2 letters from baseline in the PRP group, with a mean difference of 2.2 letters between treatment groups (*p* < 0.001) [74].

The costs of PRP versus intravitreal ranibizumab for PDR have recently been evaluated. PRP compared with intravitreal ranibizumab as primary treatment for PDR is less expensive over 2 years, but both fall well below the accepted cost per QALY upper limit [75].

Overall, these results support the intravitreal injections of ranibizumab as a possible alternative treatment for PDR.

### 3.5. Surgical Treatment of RD-Related Complications

Main indications of vitrectomy in patients with DR include tractional retinal detachment, tractional macular edema, and vitreous hemorrhage [76, 77].

Vitreous hemorrhage is one of the most frequent complications of DR. Surgical treatment is indicated for patients with DR without previous laser photocoagulation. If a previous panretinal photocoagulation has been performed, a waiting time of 3 months for reabsorption of the hemorrhage can be established, but surgery is indicated in the presence of unresolved bleeding after this interval [76, 77]. The surgical technique is usually a posterior pars plana vitrectomy with three-port sclerotomy system of 23 or 25 gauge. Endo-ocular laser during surgery can be applied. It is important to remove membranes or fibrovascular tissue that may cause retinal traction and subsequent retinal detachment. Staining of the vitreous with triamcinolone helps surgeons to achieve a complete removal of the vitreous from the retina, also acting as anti-inflammatory agent at the end of the procedure.

Pars plana vitrectomy is also the surgical option in diabetic patients with tractional retinal detachment. Technical details regarding triamcinolone injection, application of perfluorocarbon liquid, endo-ocular panretinal photocoagulation, or preoperative or perioperative anti-VEGF treatment depend on the surgeon's criteria according to individual characteristics of the patient [76, 77].

Surgical treatment of neovascular glaucoma would be indicated in the presence of new vessels and no decrease of IOP after extensive panretinal photocoagulation or intravitreal treatment with anti-VEGF drugs. Glaucoma surgery involves aqueous humor drain using different valve devices [76, 77].

Complications of vitrectomy include new hemorrhages, cataract (especially in patients over 50–55 years, which justifies combined cataract surgery and vitrectomy), and other complications as in any endo-ocular surgery such as retinal detachment and endophtalmitis [74, 75].

## 4. Concluding Remarks

DR is one of the most common microvascular complications of diabetes with the potential to cause severe vision loss and blindness and a devastating effect on quality of life. Despite a solid body of evidence regarding the importance of strict metabolic control and treatment of associated risk factors particularly hypertension, failure to maintain target HbA1c levels is a major contributing cause of development and progression of DR. Screening protocols using mydriatic and, preferable, nonmydriatic retinography should be implemented in the primary care setting. Family physicians should have adequate knowledge of the different stages of DR and the current international classification systems of DR and DME to follow recommendations for adequate screening schedules and referral, including urgency of referral to the specialized ophthalmologist. Panretinal photocoagulation should be used to treat two key complications of DR: retinal neovascularization and macular edema. Laser photocoagulation is not indicated in mild and moderate NPDR but it may be indicated in the presence of suggestive signs of development of PDR. Anti-VEGF treatment is now the first-line therapy for DME involving the central macula. Aflibercept, ranibizumab, and bevacizumab are effective antiangiogenic agents, but aflibercept is probably the most cost-effective option, with a lower cost associated with fewer intravitreal injections needed and a reduced workload in daily practice.

Fluocinolone slow release implant is effective in DME and is a promising alternative due to the reduced frequency of treatment required, but long-term follow-up data is still lacking. Patients with tractional retinal detachment, tractional macular edema, and vitreous hemorrhage are candidates for vitrectomy. In the presence of new vessels and no decrease of intraocular pressure after extensive panretinal photocoagulation or intravitreal anti-VEGF therapy, surgical treatment of neovascular glaucoma should be considered.

Finally, a fluent and robust communication between the diabetologists and the retinologists seems crucial for arresting the progression of this devastating complication of diabetes.

## Figures and Tables

**Table 1:** Recommended strategy for the control of DR taking into account the experience of the physician in performing funduscopic examinations and clinical status and comorbidities of diabetic patients.

Recommended action
(i) The doctor (e.g., general practitioner) has experience in fundus examination: systematic fundus exam to all diabetic patients at each consultation, with referral to the ophthalmologist once a year.
(ii) The doctor has no experience in fundus examination: referral to diabetic patients to the ophthalmologist after 5 years of diagnosis in type 1 diabetes and immediately after diagnosis in type 2 diabetes.
(iii) Increased controls in patients at risk: hypertension, proteinuria, dyslipidemia, and pregnancy.
(iv) Metabolic control of diabetes as strict as possible.
(v) Treatment of associated high blood pressure.
(vi) Healthy lifestyle, diet, and physical exercise.
(vii) Avoid tobacco and alcohol.

**Table 2:** Recommended periodicity of screening procedures for DR.

Recommendation	Type of diabetes	Action
Starting screening	Type 1	(i) 5 years after diagnosis
		(ii) In people older than 15 years of age
	Type 2	(i) At the time of diagnosis
	Pregnancy in a diabetic woman	(i) Before the end of the first trimester of gestation

Periodicity of screening	Type 1	(i) Annual
	Type 2 without signs of DR, adequate metabolic control, and short duration of the disease	(i) Every 2 years
	Type 2 without signs of DR, poor metabolic control or >10 years since diagnosis	(i) Every year
	Type 2 diabetes and mild NPDR	(i) Every year

**Table 3:** Recommended ophthalmological controls^∗^ in patients with DR according to stage and complications.

DR stage	Control periodicity
Nonproliferative diabetic retinopathy (NPDR)	
Mild	
Diabetic macular edema (DME)	
Present	
(i) Non-CIDME	Every 6 months
(ii) CIDME	Every 1–4 months^∗∗^
Absent	Every 12 months
Moderate	
Diabetic macular edema (DME)	
Present	
(i) Non-CIDME	Every 3-4 months
(ii) CIDME	Every 1–4 months^∗∗^
Absent	Every 6–12 months
Severe	
Diabetic macular edema (DME)	
Present	
(i) Non-CIDME	Every 3-4 months
(ii) CIDME	Every 1–4 months^∗∗^
Absent	Every 6 months
Proliferative diabetes retinopathy (PDR)	Every 3 months

^∗^In addition to optimizing blood glucose levels, lipid profile, and blood pressure. ^∗∗^In this case, intraocular treatment with anti-VEGF is recommended as first-line therapy for most eyes.

**Table 4:** Criteria and degree of urgency for referral of a patient with DR to the ophthalmologist.

Lesions requiring immediate assessment by the ophthalmologist	Proliferative retinopathy	(i) New vessels on the optic disc or at any location in the retina
		(ii) Preretinal hemorrhage
	Advanced diabetic retinopathy	(i) Vitreous hemorrhage
		(ii) Fibrotic tissue (epiretinal membrane)
		(iii) Recent retinal detachment
		(iv) Iris neovascularization

Lesions that should be referred to the ophthalmologist for assessment as soon as possible	Preproliferative retinopathy	(i) Venous irregularities
		(ii) Multiple hemorrhages
		(iii) Multiple cotton-wool exudates
		(iv) Intraretinal microvascular abnormalities (IRMA)
	Nonproliferative retinopathy with macular involvement	(i) Decreased visual acuity uncorrected with a pinhole occluder (suggestive of macular edema)
		(ii) Microaneurysms, hemorrhages, or exudates within less than one disc diameter of the center of the macula (with or without vision loss)
	Nonproliferative retinopathy without macular involvement	(i) Hard exudates with a circinate or plaque pattern in the major temporal vascular arcades
	Any other finding that the observer could not be interpreted with a reasonable degree of certainty	

Lesions requiring follow-up control (every 6–12 months) but should not be referred to the ophthalmologist	Nonproliferative retinopathy	(i) Hemorrhages or microaneurysms occasionally or hard exudates beyond one disc diameter of the center of the macula
		(ii) Isolated cotton-wool exudates without preproliferative associated lesions
